# Pilocarpine induces the residual secretion of salivary fluid in perfused submandibular glands of rats

**DOI:** 10.1371/journal.pone.0221832

**Published:** 2019-08-28

**Authors:** Takanori Narita, Bing Qi, Masataka Murakami, Hiroshi Sugiya

**Affiliations:** 1 Laboratory of Veterinary Biochemistry, Nihon University College of Bioresource Sciences, Kameino, Fujisawa, Kanagawa, Japan; 2 Department of Physiology, Nihon University School of Dentistry at Matsudo, Matsudo, Chiba, Japan; 3 Department of Nano-structure Physiology, National Institute for Physiological Sciences, Okazaki, Aichi, Japan; National Institutes of Health, UNITED STATES

## Abstract

Pilocarpine is an M3 muscarinic agonist that is widely used for the treatment of xerostomia caused by various diseases and medical conditions. Pilocarpine induced the secretion of salivary fluid in perfused submandibular glands of rats. The secretion of salivary fluid observed after removal of pilocarpine was referred to as residual fluid secretion. The volume of fluid and time of the residual secretion depended on the dose of pilocarpine. Such a residual effect of pilocarpine was observed on fluid secretion via the paracellular pathway and oxygen consumption. When a muscarinic antagonist was added to the perfusate immediately after cessation of pilocarpine, residual secretion of salivary fluid did not occur. These observations indicate that the residual secretion of salivary fluid is a characteristics of the interaction of pilocarpine with muscarinic receptors.

## Introduction

Salivary secretion is controlled by the autonomic nervous system, and occurs in response to stimulation by neurotransmitters released from nerve endings [1, 2]. Simulated parasympathetic neurons primarily release acetylcholine, which binds to muscarinic cholinergic receptors in the acinar cells of salivary glands. The activation of these muscarinic cholinergic receptors leads to an increase in intracellular calcium ion levels, which induces copious fluid secretion via aquaporin channels located on the plasma membrane of acinar cells (transcellular pathway), or via the tight junction complex of adjacent acinar cells (paracellular pathway) [1–6]. In contrast, stimulated sympathetic nerves release norepinephrine, which activates β-adrenergic receptors and provokes high levels of protein secretion [1, 5].

Salivary hypofunction results in xerostomia (dry mouth), which is caused by numerous medical conditions including in patients who receive therapeutic irradiation to treat head and neck cancer, autoimmune diseases such as Sjögren’s syndrome, graft-versus-host disease and as side effect of using certain medications [2, 7]. Long-standing xerostomia leads to oral infections such as dental caries and periodontitis, and difficulty speaking, chewing, and swallowing [2, 7]. For relief of xerostomia symptoms, muscarinic agonists are clinically used as sialagogues.

Pilocarpine, a natural plant alkaloid derived from the leaves of *Pilocarpus jaborandi*, is a muscarinic agonist. It binds to M3 muscarinic receptors in salivary glands and increases the salivary secretion for an adequate length of time; in addition, pilocarpine can be administered by mouth and has tolerable side effects [8, 9]. Therefore, this drug has been widely used clinically for the treatment of xerostomia caused by various diseases and medical conditions including Sjögren’s syndrome [10–15] and radiation-induced xerostomia after radiotherapy for head and neck cancer [16–19].

It is well accepted that systemic administration of pilocarpine induces salivation not only by activation of central mechanisms [20–24], but also by acting directly on salivary glands [25–27]. To evaluate the direct effects of neurotransmitters and drugs on salivary flow and secretion, perfused submandibular glands of rats were used as the closest *in vitro* model for the in vivo situation [28–31]. In this study, we demonstrate that pilocarpine induces the residual secretion of salivary fluid, salivary fluid secretion continued after the cessation of pilocarpine treatment, in perfused submandibular glands of rats.

## Materials and methods

### Materials

Pilocarpine hydrochloride was purchased from Wako Pure Chemical Industries (Osaka, Japan). Tetrodotoxin (TTX) was obtained from Funakoshi (Tokyo, Japan). Carbamylcholine chloride (carbachol) was purchased from Tokyo Kasei (Tokyo, Japan). 1,1-Dimethyl-4-diphenylacetoxypiperidinium iodide (4-DAMP) was purchased from Tocris Bioscience (Ellisville, MI). Collagenase and bovine serum albumin (BSA) were purchased from Roche Diagnostics (Basel, Switzerland). Mouse monoclonal anti-occuludin and rabbit polyclonal anti-aquaporin-5 antibodies were obtained from Zymed Laboratories (San Francisco, CA) and Chemicon (Temecula, CA), respectively. Alexa Fluor-488-labeled and Alexa Fluor-568-labeled secondary antibodies, TO-PRO-3, and Lucifer yellow (LY) were purchased from Molecular Probes (Eugene, OR).

### Preparation of perfused submandibular glands of rats

Perfused submandibular glands were prepared as previously described [30]. Briefly, Wistar male rats (250–350 g) were anesthetized with pentobarbitone sodium (50 mg/kg body weight) by intraperitoneal injection, and submandibular and sublingual glands were surgically isolated from the rats. The branches of the feeding artery and draining vein were ligated, and the attached sublingual glands were removed. A 0.3 mm I.D. × 0.5 mm O.D. tube (EXLON^TM^, Iwase, Kanagawa, Japan) was cannulated into the extralobular main duct from each submandibular gland for sampling. A stainless-steel catheter (26G) connected to the infusion line for perfusion was cannulated into the artery distal to the glandular branch. The vein from the gland was cut freely. The gland was placed on an organ bath (37°C), and the arterial catheter was connected to the perfusion apparatus. The venous effluent was continually drained. Arterial perfusion of the glands was performed at a rate of 2 mL/min using a peristaltic pump (Cole-Palmer, Barrington, IL) to supply enough oxygen without a specific oxygen carrier during the secretory phase. The composition of perfusate was as follows (in mM): NaCl, 145; KCl, 4.3; CaCl_2_, 1; MgCl_2_ 1; glucose, 5; and 10 mM HEPES (pH 7.4). The perfusate was equilibrated with 100% O_2_. Agonists and antagonists used were dissolved in the perfusate. The rats were euthanized immediately after removing the glands by intraperitoneal administration with pentobarbitone sodium (100 mg/kg body weight). All animal protocols were approved by the Laboratory Animal Committee of the Nihon University School of Dentistry at Matsudo (#08–0049).

### Measurement of secreted fluid

The secreted fluid was quantified as follows: The tip of the ductal cannula filled with pefusate was put, below the surface of the water, in a cup filled with water on an electronic balance (Shimadzu AEG-220, Kyoto, Japan), avoiding any contact with the bottom of the cup. The cumulative weight of the salivary fluid secreted was automatically measured every 3 s and was transferred to a computer. The cumulative weight was assumed to be the cumulative volume of saliva assuming a saliva specific gravity of 1. The rate of fluid secretion was calculated from the time-differentiation of the cumulative volume.

### Lucifer yellow (LY) measurement

Fluid secretion via the paracellular pathway was determined using LY [3, 30].

The perfusate containing LY (500 nM) was perfused, and salivary fluid was collected at 1 min intervals. A DTX 800 Fluorescence Reader (Beckman Coulter, Brea, CA) was used for the measurement of the fluorescence intensity of LY in the inflow perfusate and in the salivary fluid. The fluorescence intensity in the salivary fluid is dependent on the fluorescence in the inflow perfusate. Therefore, the amount of LY transported in the salivary fluid expressed as the perfusate clearance of LY by the submandibular glands was calculated using the following equation:

SLY=(CS/CIP)×F/submandibularglandweight

where SLY (μL/min/g) is the flow rate of LY thorough the paracellular pathway; F is the flow rate of salivary fluid secreted per min; CS is the concentration of LY in salivary fluid collected per min; and CIP is the concentration of LY in the perfusate. Results were normalized to weight of the submandibular gland (g).

### Measurement of oxygen consumption

The polarographical measurement of the partial oxygen pressure of the perfusate and effluent was performed by means of Clark-type electrodes (Instech Laboratories, Plymouth Meeting, PA) at 37°C. The electrodes were placed sequentially on the arterial and venous sides of the perfusion line. The oxygen consumption rate was calculated from the arteriovenous difference in oxygen content and the perfusion rate, and was determined from data of the O_2_ solubility, with suitable corrections for atmospheric pressure and H_2_O vapor pressure, as previously reported [29, 30].

### Immunohistochemistry

Submandibular glands were immediately frozen in liquid nitrogen after perfusion with pilocarpine. Cryostat sections (5 μm) were fixed in ice-cold methanol for 15 min and rinsed in phosphate-buffered saline (PBS, 3 × 10 min each). Sections were incubated with anti-aquaporin-5 (1:100) and anti-occludin (1:50) antibodies overnight at 4°C. The sections were washed several times in PBS, and incubated with the respective Alexa Fluor-labeled secondary antibodies for 1 h at room temperature. Nuclei were stained with TO-PRO-3 (1:1000). Fluorescent images were acquired using an LSM-510 META (Carl Zeiss, Oberkochen, Germany) confocal microscopy.

### Statistical analysis

The statistical significance differences between values in the absence and presence of pilocarpine were determined by ANOVA. When the effect of tetrodotoxin on secretion of salivary fluid was examined, double-tailed *t*-test was used. *P* values regarded as statistically significant were indicated by asterisks: *, *P* < 0.05, **, *P* < 0.01, and ***, *P* < 0.001.

## Results and discussion

### Effect of pilocarpine on secretion of salivary fluid

**Fig 1:**
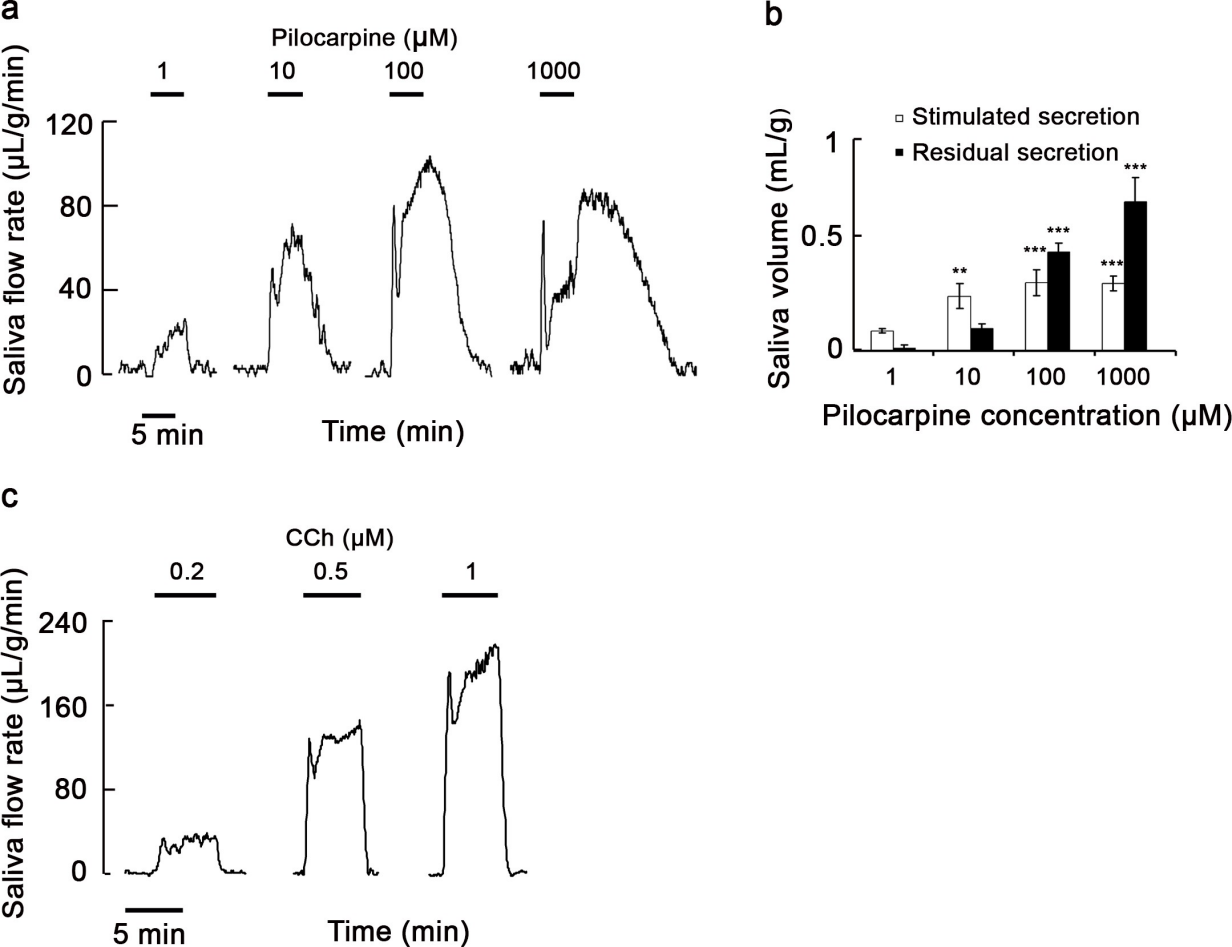
Presence of residual secretion of salivary fluid induced by pilocarpine in perfused submandibular glands of rats. (a, c) Profile of residual secretion of salivary fluid induced by pilocarpine (a) or carbachol (c). Submandibular glands were stimulated with 1–1000 μM pilocarpine or 0.2–1 μM carbachol (CCh) for 5 min. Stimulating periods are indicated as black bars. Results are representative of three independent experiments. (b) Stimulated and residual salivary fluid secretion induced by various concentrations of pilocarpine. Data are means ± S.E. of three independent experiments. **, *P* < 0.01 and ***, *P* < 0.001 compared with unstimulated glands.

We examined the effect of pilocarpine on secretion of salivary fluid in perfused submandibular glands of rats. As shown in Fig 1A and 1B, 1–1000 μM pilocarpine induced secretion of salivary fluid, whereas less secretion of salivary fluid occurred in the absence of pilocarpine. The pattern of fluid secretion during stimulation consisted of two phases, a sharply transient phase and a subsequent sustained phase. Interestingly, after ceasing the stimulation with pilocarpine, salivary fluid secretion continued and gradually returned to the baseline, which we referred as residual fluid secretion. Salivary fluid secretion in the transient, sustained, and residual phases increased by 1–1000 μM pilocarpine in a dose-dependent manner. The fluid secretion rate in the transient and sustained phases plateaued at 100 μM, but the residual rate did not. Conversely, this residual secretion of salivary fluid was not observed when the glands were stimulated with the muscarinic agonist carbachol (Fig 1C).

**Fig 2:**
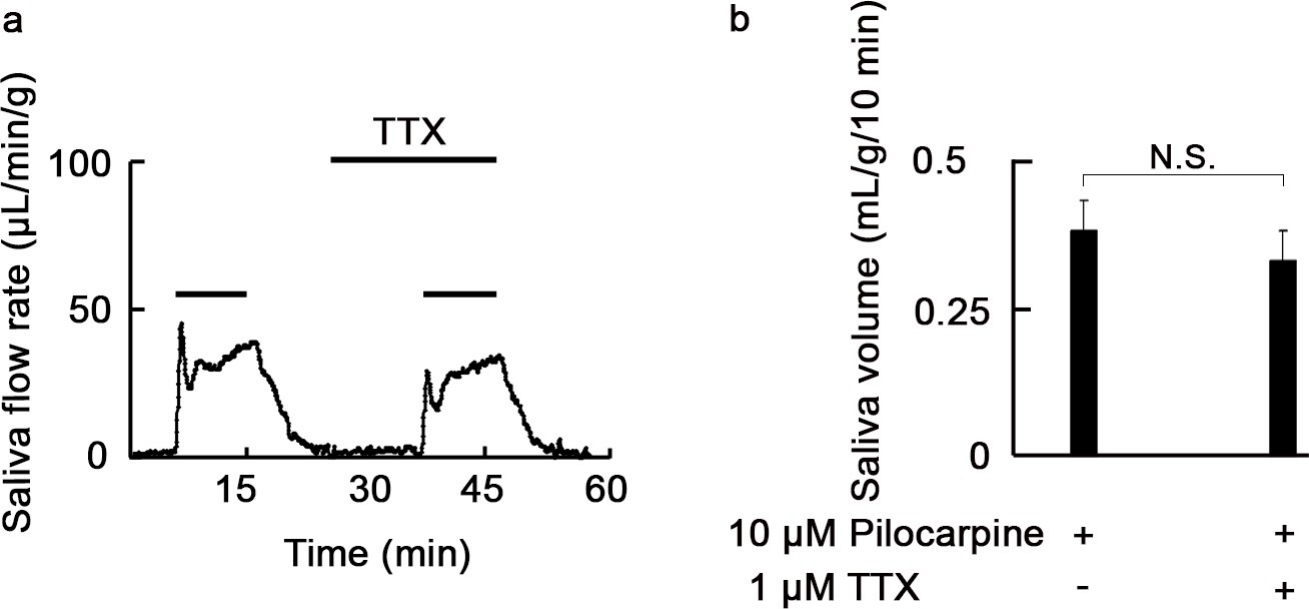
Tetrodotoxin has no effect on pilocarpine-induced fluid secretion in perfused submandibular glands of rats. (a) Profile of secretion of salivary fluid in response to pilocarpine treatment in the presence or absence of tetrodotoxin (TTX) in submandibular glands of rats. Submandibular glands were stimulated with 10 μM pilocarpine in the absence of TTX. After removal of pilocarpine, the glands were incubated with 1 μM tetrodotoxin for 10 min and subsequently stimulated with 10 μM pilocarpine in the presence of TTX. Presence of drugs is indicated by the black bars. Results are representative of three independent experiments. (b) Pilocarpine-induced salivary fluid volume in the presence or absence of TTX in rat submandibular glands. Data are means ± S.E. of three independent experiments.

The submandibular glands used in this study contain some intrinsic neuron fibers. To exclude the effect of neurotransmitters released from neurons in the glands, the effect of pilocarpine on salivary fluid secretion was examined in the presence of tetrodotoxin, a specific blocker of the voltage-gated Na^+^-channels in nerve cell membranes. However, tetrodotoxin had no effect on pilocarpine-induced salivary fluid secretion, including residual secretion (Fig 2), indicating that pilocarpine directly stimulates submandibular glands in our perfusion system.

**Fig 3:**
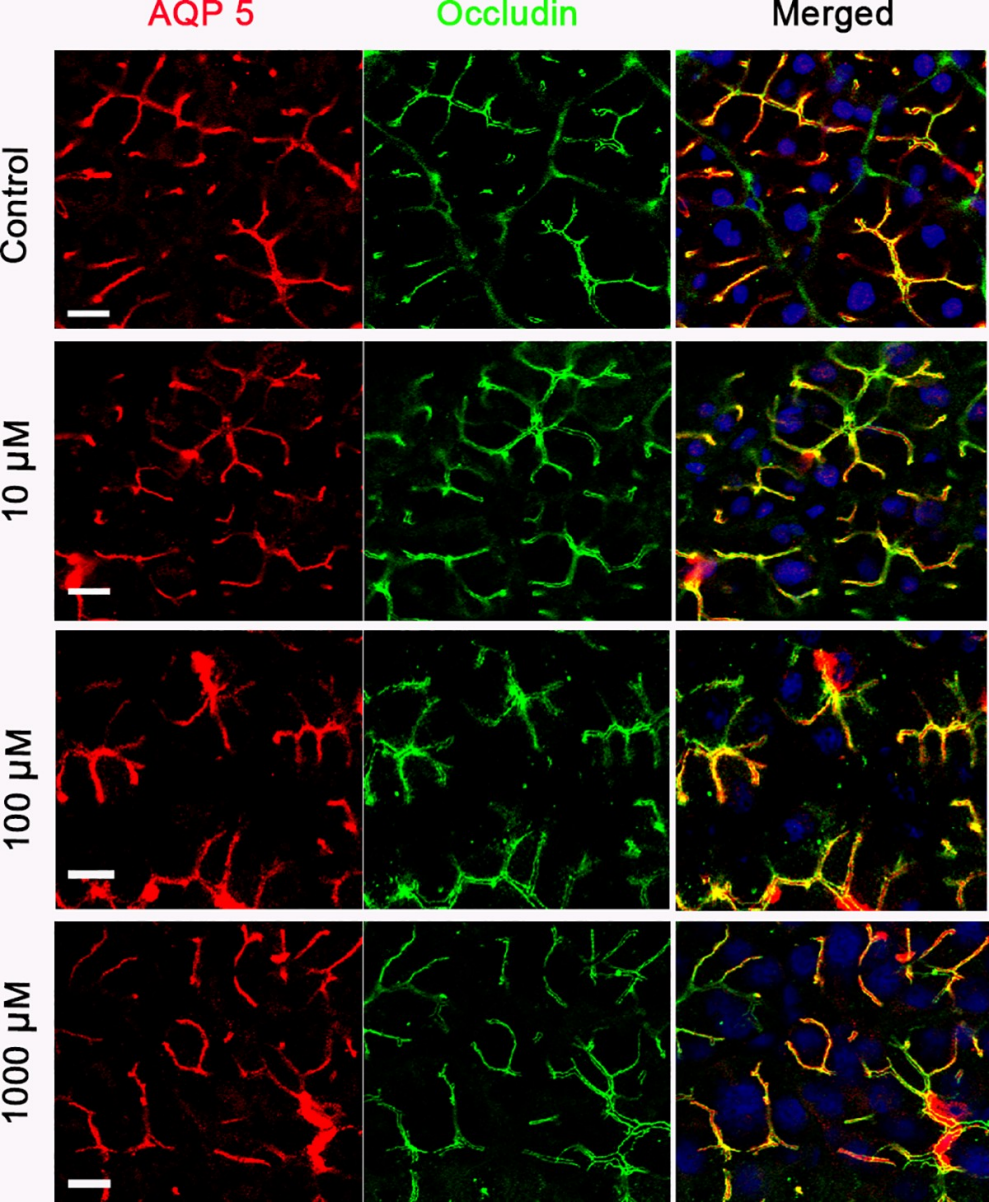
Pilocarpine stimulation had no effect on the subcellular structures of perfused submandibular glands of rats. Submandibular glands were perfused with perfusate containing 0 (Control) -1000 μM pilocarpine for 5 min, and were sectioned by cryostat. Sections were stained with anti-aquaporin 5 conjugated with Alexa Fluor 568 (red), anti-occludin conjugated with Alexa Fluor 488 (green), and TO-PRO3 to detect the nuclei (blue). Results are representative of three independent experiments.

In humans, plasma concentrations of under μM range of pilocarpine has been reported to induce salivation [32]. However, we used much higher doses of pilocarpine to induce salivary fluid secretion including the residual secretion. To exclude the cytotoxic effects of pilocarpine, we performed light microscopic studies. Consequently, the morphology of the rat submandibular glands stimulated with 1 mM pilocarpine did not differ from that of control (S1 Fig). Furthermore, we examined the localization of the water channel aquaporin 5 (AQP5) and occludin, which are integral plasma-membrane proteins located at the apical membranes and the tight junctions of acinar cells [33, 34], respectively, in the glands stimulated with pilocarpine. Nevertheless, 10–1000 μM pilocarpine had no effect on the localization of both membrane proteins (Fig 3), indicating that the effect of pilocarpine on salivary fluid secretion is distinct from the cytotoxic effects of the drug.

### Lucifer yellow (LY) secretion

**Fig 4:**
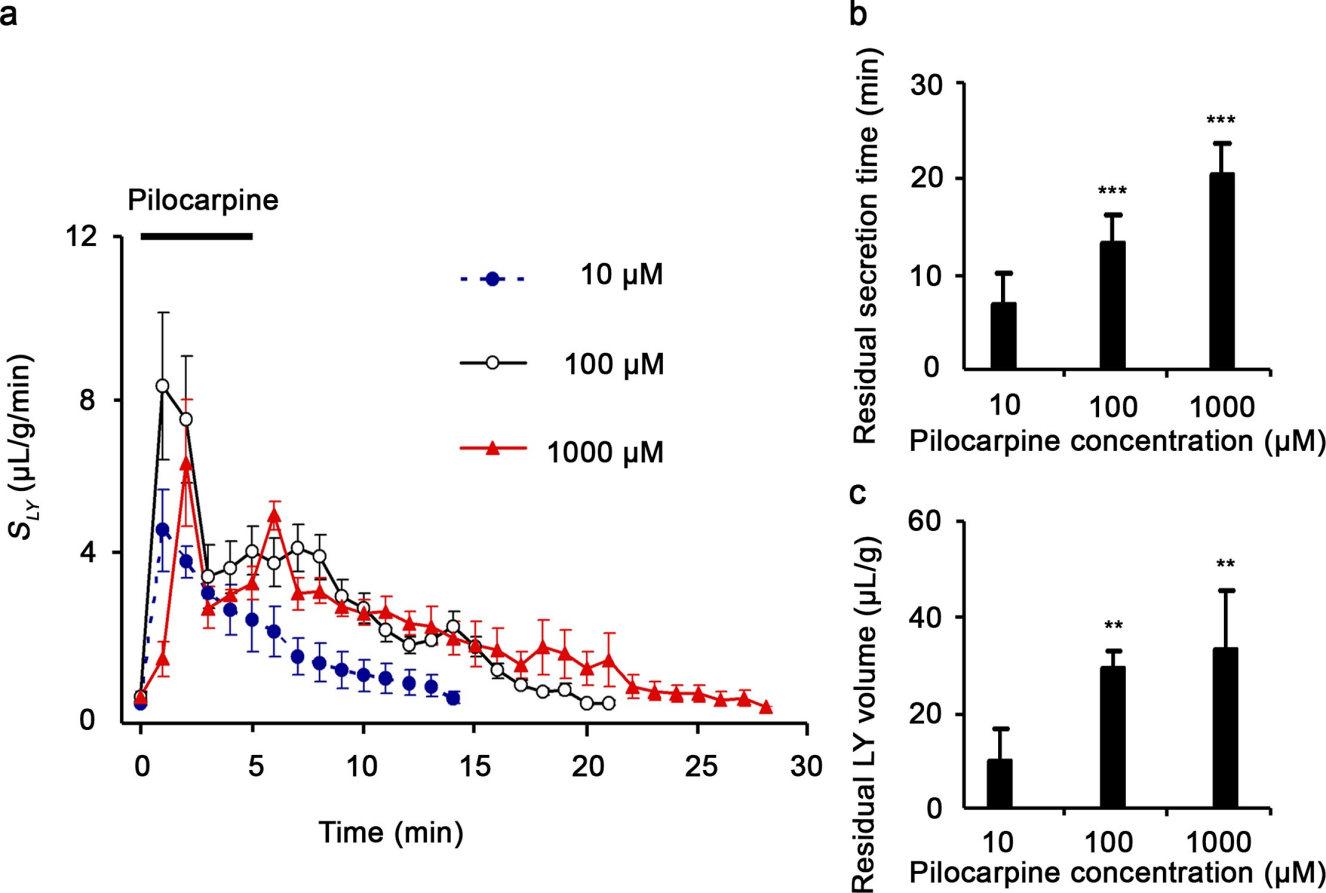
Residual paracellular secretion induced by pilocarpine in perfused submandibular glands of rats. Profile of pilocarpine-induced Lucifer Yellow (LY) secretion in perfused submandibular glands of rats (a). Submandibular glands were stimulated with 10–1000 μM pilocarpine for 5 min, and LY in secreted saliva was determined. Stimulation time is depicted as a black bar. Results are representative of three independent experiments. Dose-dependent effect on time (b) and volume (c) of pilocarpine-induced LY secretion. Data are means ± S.E. of three independent experiments. **, *P* < 0.01 and ***, *P* < 0.001 compared with unstimulated glands.

Secretion of salivary fluid is caused by fluid transport via two pathways, in which fluid is moved across the plasma membrane of cells and between cells [1–6]. In previous studies, LY, a cellular impermeable substance, was used as an effective tracer that detects fluid transport via the paracellular pathway [3, 30]. Thus, we examined the effect of pilocarpine on LY secretion. After stabilization with perfusate containing 500 nM LY, the submandibular glands were stimulated with 10–1000 μM pilocarpine. In the glands stimulated with pilocarpine, LY secretion with residual phase was observed (Fig 4A). The time and volume of the residual secretion of LY increased by 10–1000 μM pilocarpine in a dose-dependent manner (Fig 4B and 4C, respectively). These results suggest that the fluid secreted via the paracellular pathway is included in the residual salivary fluid secreted from the glands stimulated with pilocarpine.

### Oxygen consumption during pilocarpine-induced fluid secretion

**Fig 5:**
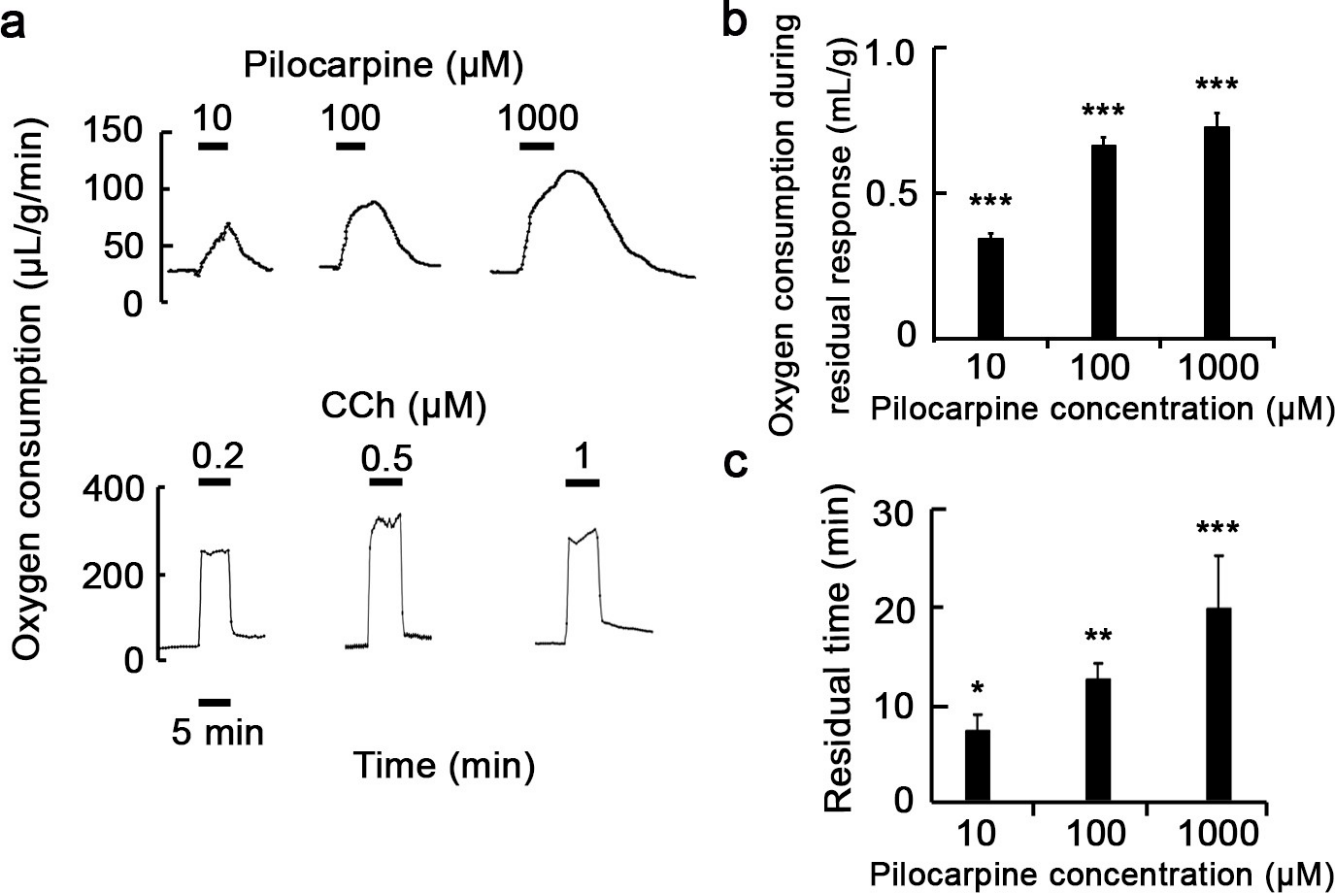
Pilocarpine-induced residual oxygen consumption in perfused submandibular glands of rats. Submandibular glands were stimulated with 1–1000 μM pilocarpine or 0.2–1 μM carbachol (CCh) for 5 min. Profiles of oxygen consumption induced by pilocarpine or carbachol (a). Stimulating duration is indicated as black bars. Results are representative of three independent experiments. Residual oxygen consumption (b) and residual time (c) induced by various concentrations of pilocarpine. Data are means ± S.E. of three independent experiments. *, *P* < 0.05, **, *P* < 0.01 and ***, *P* < 0.001 compared with unstimulated glands.

It has been reported that the increase in oxygen consumption during pilocarpine-induced fluid secretion is induced by secretagogues in submandibular glands, suggesting that most of the oxygen consumption is utilized for transport of ions and water [29, 35]. Therefore, we investigated oxygen consumption during fluid secretion induced by pilocarpine. After stabilization with perfusate saturated by O_2_, submandibular glands were stimulated with 10–1000 μM pilocarpine. As Fig 5A shows, pilocarpine induced a prompt increase in oxygen consumption in a dose-dependent manner with a residual effect. The total oxygen consumption and the residual time of oxygen consumption after removal of pilocarpine increased, and both were dependent on the dose of pilocarpine used (Fig 5B and 5C). In contrast, in submandibular glands stimulated with 0.2–1 μM carbachol, such a residual effect of pilocarpine was not observed (Fig 5A). Taken together, it is likely that the residual effect is a specific secretory function induced by pilocarpine.

### Effect of muscarinic receptor antagonist for pilocarpine-induced residual secretion of salivary fluid

**Fig 6:**
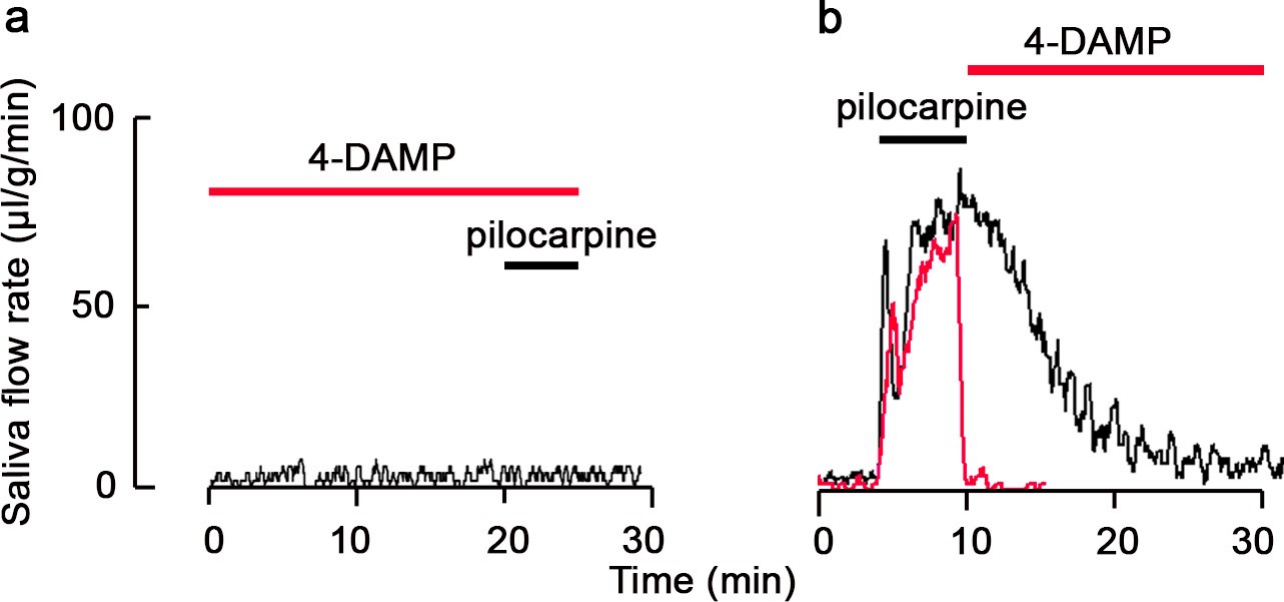
Effect of the muscarinic antagonist 4-DAMP on pilocarpine-induced residual secretion of salivary fluid in perfused submandibular glands of rats. (a) Effect of 4-DAMP on pilocarpine-induced salivary fluid secretion. After pre-incubation with 100 μM 4-DAMP, submandibular glands were stimulated with 100 μM pilocarpine. (b) After stimulation with 100 μM pilocarpine, submandibular glands were perfused with 100 μM 4-DAMP with pilocarpine. Periods of 4-DAMP treatment and pilocarpine stimulation are depicted as red and black bars, respectively. Results are representative of three independent experiments.

To elucidate involvement of muscarinic receptors in the residual secretion of salivary fluid, we examined the effect of the muscarinic receptor antagonist 4-DAMP on pilocarpine-induced salivary fluid secretion. When submandibular glands were pretreated with 4-DAMP (100 μM) and stimulated with pilocarpine (100 μM), pilocarpine failed to induce salivary fluid secretion (Fig 6A). However, when the submandibular glands were treated with 4-DAMP after stimulation with pilocarpine, pilocarpine-induced residual fluid secretion was completely abolished (Fig 6B). These results indicate that muscarinic receptor activation contributes to pilocarpine-induced residual secretion of salivary fluid.

In Chinese hamster ovary cells expressing the M3 muscarinic receptors, the association and dissociation rate constants of the muscarinic agonists were measured using a *l*-[*N*-methyl]-[^3^H]scopolamine methyl chloride competition binding assay, and the association and dissociation rates of pilocarpine were higher and lower than that of carbachol, respectively [36]. Further, the efficacy of muscarinic agonists has been demonstrated to be positively correlated with the rate of dissociation from the M3 receptor. Therefore, it is likely that the residual secretion of salivary fluid is caused by the residency of pilocarpine on M3 receptors of the submandibular gland. On the other hand, pilocarpine has been demonstrated to provoke salivary fluid secretion by activating a mixture of M1 and M3 muscarinic receptors, because salivary secretion induced by high doses of pilocarpine was abolished in M1/M3 receptor double-KO mice, but not in in M1 and M3 receptor single-KO mice [37]. Furthermore, 4-DAMP has been demonstrated to exhibit equivalent high affinities for the M1 and M3 muscarinic receptors [38, 39]. Therefore, the interaction between M1 and M3 receptors appears to be involved in pilocarpine-induced residual response, although we need further studies.

In this study, we demonstrated that pilocarpine induced the residual secretion of salivary fluid in perfused submandibular glands of rats. Such an effect of pilocarpine was observed in the submandibular glands of mice [26]. However, in clinical studies, the salivary secretory rate in patients treated with pilocarpine gradually returns to baseline after the discontinuation of the drug [40, 41]. Further, the amount of stimulated and unstimulated saliva in patients treated with pilocarpine has also been demonstrated to be higher than the initial baseline before treatment, which was described as a carryover effect of pilocarpine [42]. It has been noted that the threshold of salivary stimulation on muscarinic receptors may be reduced by long-term exposure to pilocarpine [40], or pilocarpine may enhance acetylcholine release by preganglionic stimulation [43], although the precise mechanisms are obscure. It likely that the residual response of pilocarpine on salivary glands contributes to the systemic effect of pilocarpine.

## Conclusions

In conclusion, pilocarpine provoked residual secretion of salivary fluid in perfused submandibular glands of rats. The effect of pilocarpine is caused by the residency of pilocarpine on muscarinic receptors in the submandibular gland.

## Supporting information

S1 Fig Histological examination by hematoxylin and eosin staining. After perfusion with normal or pilocarpine-containing perfusate, the submandibular glands were immediately fixed in neutral phosphate buffered 3.7% formalin. The tissue sections from the formalin-fixed tissues were stained with hematoxylin and eosin for histology observation. (a) Control and (b) 1 mM of pilocarpine-perfused rat submandibular glands. Scale bar = 20 μm.
(TIF)
