# Supplementary figures and images for: Pilocarpine induces the residual secretion of salivary fluid in perfused submandibular glands of rats
Source: PLoS One. 2019 Aug 28;14(8):e0221832. doi: 10.1371/journal.pone.0221832 (PMC6713442; doi:10.1371/journal.pone.0221832)

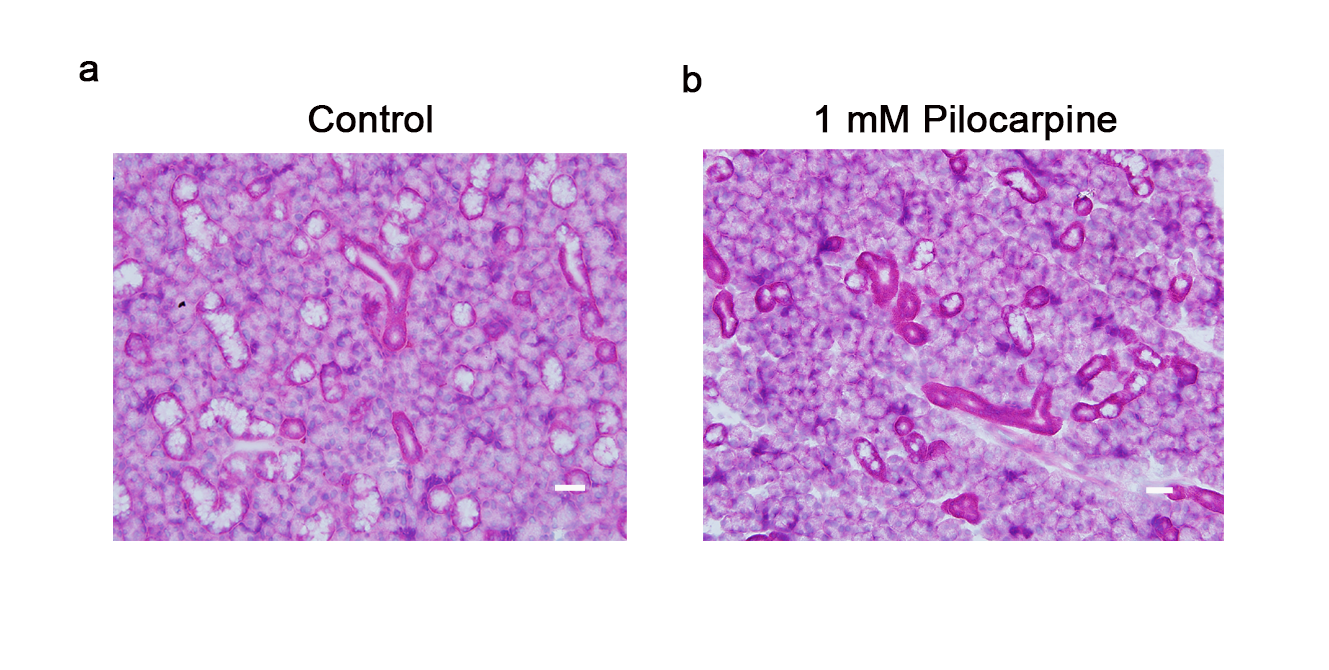

Supplement: S1 Fig — After perfusion with normal or pilocarpine-containing perfusate, the submandibular glands were immediately fixed in neutral phosphate buffered 3.7% formalin. The tissue sections from the formalin-fixed tissues were stained with hematoxylin and eosin for histology observation. (a) Control and (b) 1 mM of pilocarpine-perfused rat submandibular glands. Scale bar = 20 μm. (TIF) [file pone.0221832.s001.tif]
